# Selective extraocular muscle regression following tocilizumab in active thyroid associated ophthalmopathy: a 3D MRI-based retrospective study

**DOI:** 10.3389/fendo.2026.1930592

**Published:** 2026-08-26

**Authors:** Fang Nan, Yuehui Zhang, Yan Ma, Cuixia Yang, Xiumei Ma, Ningyu An, Shunyu Piao, Xiao Zhang, Li Yang, Yang Zheng, Xiaomin Cui, Ting Ma, Qifan Mai, Wenjuan Zhuang, Qian Li

**Affiliations:** 1 Department of Ophthalmology, Peoples’ Hospital of Ningxia Hui Autonomous Region, The Third Affiliated Clinical College of Ningxia Medical University, Yinchuan, Ningxia, China; 2 Department of Ophthalmology, General Hospital of Ningxia Medical University, Xingqing District, Yinchuan, Ningxia, China; 3 Ningxia Aier Eye Hospital, Jinfeng District, Yinchuan, Ningxia, China

**Keywords:** cross-sectional area (CSA), extraocular muscle (EOM), selective reduction, thyroid-associated ophthalmopathy (TAO), tocilizumab (TCZ)

## Abstract

**Background:**

Thyroid-associated ophthalmopathy (TAO) is an autoimmune orbital disorder characterized by orbital inflammation and hyperplasia of extraocular muscles (EOMs) and adipose tissue. Tocilizumab (TCZ) has demonstrated anti-inflammatory effects in TAO, but its ability to induce structural regression of hypertrophic EOMs has not been objectively quantified using advanced three-dimensional (3D) imaging techniques. This retrospective study aimed to evaluate the clinical and structural effects of TCZ in active moderate-to-severe TAO using quantitative 3D MRI.

**Methods:**

Twenty-four patients with active moderate-to-severe TAO who completed a standard four-dose TCZ regimen (8 mg/kg every four weeks) were enrolled. Clinical parameters (Clinical Activity Score (CAS), orbital tension, proptosis) and serum biomarkers (thyrotropin receptor antibody (TRAb), interleukin-6 (IL-6) were assessed at baseline (T1), post-treatment (T2), and at one-month follow-up (T3). Structural remodeling was quantified by measuring the cross-sectional areas (CSA) of individual EOMs and orbital fat volume (OFV) using coronal 3D modified Dixon quantitative T1-weighted MRI.

**Results:**

TCZ induced rapid inflammatory inactivation, with median CAS declining from 3 to 0 (*P* < 0.001). Proptosis showed modest reduction (20.00 mm to 19.75 mm, *P* = 0.020). Quantitative MRI revealed the most pronounced CSA reduction in the superior rectus (*P* < 0.001), followed by significant decreases in the inferior and lateral recti (*P* < 0.05), whereas no significant changes were observed in the medial rectus CSA (*P* = 0.170) or OFV (*P* = 0.179). Serum TRAb significantly decreased (4.95 to 4.62 IU/L, *P* = 0.007), while IL-6 showed a modest increase (1.50 to 2.98 pg/mL, *P* = 0.070). TCZ was well tolerated, with no severe adverse events observed. Most patients maintained clinical improvement at one-month follow-up without rebound.

**Conclusions:**

TCZ is a rapid, potent, and safe intervention for active moderate-to-severe TAO. Quantitative 3D MRI provides objective evidence that TCZ selectively induces structural regression of hypertrophic EOMs, with the superior rectus showing the most pronounced response and the medial rectus exhibiting considerable resistance, which may reflect chronic structural remodeling or fibrosis-associated changes. The significant decline in serum TRAb levels further supports the systemic immunomodulatory effects of TCZ. These findings provide imaging-based evidence for the differential structural effects of TCZ on orbital tissues.

## Introduction

Thyroid-associated ophthalmopathy (TAO) is the most common extrathyroidal manifestation of Graves’ disease (1, 2). This condition is a complex autoimmune inflammatory disorder characterized by the pathological expansion of orbital fat and connective tissue. Edematous hypertrophy of the extraocular muscles (EOMs), driven by hyaluronan accumulation and inflammatory infiltration, is a hallmark of the disease (3). Patients frequently experience symptoms including eyelid retraction, proptosis, and diplopia. In severe cases, they may develop dysthyroid optic neuropathy (DON), which significantly compromises quality of life (4).

Intravenous glucocorticoids (IVGCs) are currently the standard first-line therapy for patients with active moderate-to-severe TAO, as outlined in the 2021 EUGOGO guidelines (5). Nevertheless, approximately 20–30% of patients show a suboptimal response or develop resistance to steroid therapy (6). Furthermore, the potential systemic adverse effects associated with high-dose glucocorticoids (GCs) underscore the need for targeted biological alternatives (7).

Recent studies have suggested that interleukin-6 (IL-6) is a key driver of orbital fibroblast activation and the expression of Thyroid-Stimulating Hormone (TSH) receptors (8). These findings provide a strong rationale for the use of Tocilizumab (TCZ), a humanized IL-6 receptor antagonist (9, 10). The randomized clinical trial conducted by Perez-Moreiras et al. (11) provided important clinical evidence supporting TCZ, demonstrating that it significantly improved the Clinical Activity Score (CAS) and reduced proptosis in patients with TAO. Subsequent real-world studies have further corroborated its efficacy in inducing clinical remission in refractory cases (12, 13).

Although TCZ effectively inhibits IL-6 signaling in OFs, it remains unclear whether it affects individual EOMs and orbital fat volume (OFV). Most existing studies have relied on conventional imaging techniques, which lack the sensitivity required to precisely quantify structural changes in individual EOMs and orbital adipose tissue (9, 14, 15). Therefore, the structural remodeling effects of TCZ on orbital tissues warrant objective evaluation using high-resolution quantitative imaging techniques.

We retrospectively analyzed 24 patients with active moderate-to-severe TAO who received TCZ treatment. Using 3D mDixon-Quant MRI, we precisely measured the cross-sectional areas (CSA) of individual EOMs and OFV and integrated these metrics with clinical parameters and serological biomarkers (TRAb and IL-6) to characterize the structural remodeling effects of TCZ on orbital tissues.

## Materials and methods

### Study design

This retrospective, single-arm, observational study was conducted at the Peoples’ Hospital of Ningxia Hui Autonomous Region and adhered to the Declaration of Helsinki. The study protocol was approved by the Ethics Committee (Approval No. 2026-LL-152), and the requirement for informed consent was waived due to the retrospective nature of the study. We reviewed the medical records of patients diagnosed with TAO who received TCZ between January 1, 2023, and December 31, 2025. The diagnosis and classification of moderate-to-severe active TAO were based on the 2021 EUGOGO guidelines (5).

The inclusion criteria were as follows: (1) age ≥ 18 years; (2) CAS > 3/7 (5, 16); (3) moderate-to-severe disease (Class 3–6 according to NOSPECS); and (4) steroid-naive or steroid-resistant status (failure to achieve a CAS reduction of > 2 points after steroid treatment despite a cumulative methylprednisolone dose > 4.5 g, or relapse/adverse events precluding further steroid use).

Patients with a history of orbital decompression or radiotherapy, contraindications to TCZ (including active systemic infections or severe hepatic dysfunction with ALT/AST > 1.5× the upper limit of normal), or hematological abnormalities (NEUT < 2.0×10^9^/L or PLT < 100×10^9^/L) were excluded. Pregnant and lactating women were also excluded.

**Figure 1:**
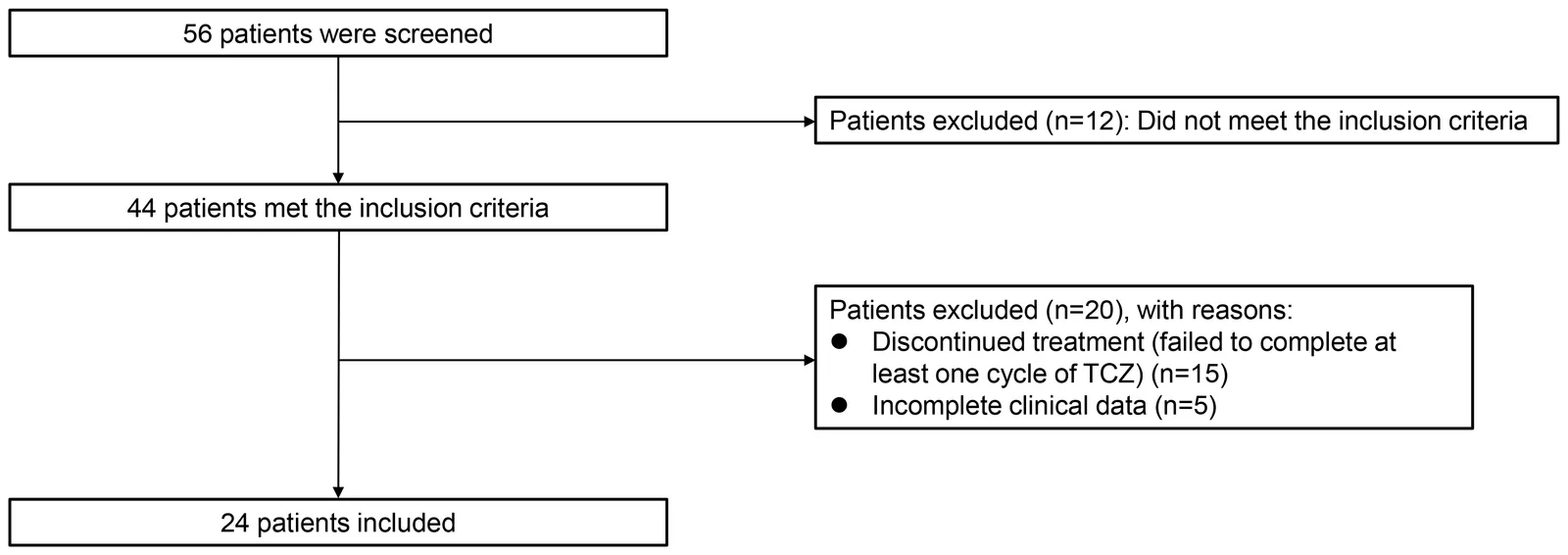
Inclusion and exclusion of patients with active TAO. A total of 56 patients with active moderate-to-severe TAO were initially screened. After application of the inclusion and exclusion criteria, 32 patients were excluded for the following reasons: 12 did not meet the diagnostic criteria, 15 did not complete the full 4-dose TCZ treatment regimen, and 5 had incomplete clinical or radiographic data. Consequently, 24 patients were included in the final analysis.

Of the 56 patients screened, 24 met the eligibility criteria and were enrolled. The 32 excluded patients comprised 12 who did not meet the diagnostic criteria, 15 who did not complete treatment, and 5 with incomplete data (**Figure 1**). Data were collected at three time points: baseline (T1), post-treatment (T2), and one-month follow-up (T3). All patients continued clinical follow-up after T3 to assess long-term stability.

### Treatment protocol

Patients received intravenous tocilizumab (TCZ, Actemra^®^, Roche Pharma, Switzerland) at a dose of 8 mg/kg, with a maximum dose of 800 mg per infusion, administered every 4 weeks for a total of four consecutive cycles.

The calculated dose of TCZ was diluted in 250 mL of 0.9% sterile saline and administered intravenously over 1 hour. Vital signs were monitored during the infusion, and patients were observed for an additional 30 minutes after the infusion to detect any acute hypersensitivity reactions.

Safety surveillance was rigorously maintained throughout the study. Before the initial dose, all patients underwent screening for hepatitis markers. Mandatory laboratory evaluations, including complete blood count, liver function tests, and coagulation profiles, were performed before each of the four infusions to monitor for systemic toxicity throughout the treatment period.

### Clinical and radiographic assessment

Clinical parameters were retrieved from medical records using a standardized case report form. All assessments were performed by a single experienced ophthalmologist at T1, T2, and T3 to ensure intra-observer consistency. Data extraction was conducted independently by two trained research assistants, and discrepancies were resolved by consensus. No imputation was performed for missing data; cases with incomplete key variables were excluded (**Figure 1**).

The 7-point CAS was used to define disease activity, with CAS < 3 indicating the inactive phase and CAS ≥ 3 indicating the active inflammatory state. Disease severity was evaluated using the NOSPECS classification. In asymmetric cases, the more severely affected eye was selected for analysis. Exophthalmos was measured using Hertel exophthalmometry, and orbital tension was assessed by digital palpation.

Serum biomarkers included IL-6 and the thyroid function panel (FT3, FT4, TSH, TRAb, TPOAb, TgAb). Safety was assessed using complete blood counts (WBC, NEUT, LYM, NEUT%, LYM%, HGB, PLT) and liver function tests (ALT, AST, TBIL, DBIL, IBIL, GGT, ALP) at each visit. Lower extremity venous ultrasonography was performed to screen for DVT.

MRI scans were performed at T1 and T2 using a 3.0-T scanner (Ingenia, Philips Healthcare) with an 8-channel head-neck coil (17–19). The 3D mDixon-Quant T1WI sequence parameters were as follows: repetition time, 7 ms; echo time, 2 ms; flip angle, 10°; field of view, 120 × 150 mm²; matrix, 320 × 254; and slice thickness, 0.9 mm (17, 18). MRI was not performed at T3 to avoid additional financial burden, as imaging at T1 and T2 was considered sufficient for evaluating structural improvement.

Quantitative analysis was performed by two independent radiologists blinded to the clinical data. (1) EOM assessment. The study eye was selected based on the greatest cross-sectional EOM involvement on MRI. Although volumetric measurement theoretically provides a comprehensive assessment of total muscle volume, accurate 3D segmentation of EOMs in active TAO is often highly susceptible to interference due to severe apical crowding, resulting in substantial measurement errors. Therefore, we utilized the maximal CSA on coronal water-phase images as a more practical and highly reproducible surrogate indicator to ensure data accuracy (14, 15, 20). CSA was measured on coronal water-phase images using OsiriX MD (v. 12.0). Muscle selection followed the hierarchy of IR, MR, SR, and LR (15). To ensure rigorous methodological reproducibility across serial examinations, the selection of the measurement slice was standardized using fixed anatomical landmarks. The radiologists tracked the EOMs on consecutive coronal slices along the optic nerve axis, starting from the posterior pole of the globe and extending posteriorly to the orbital apex. Through this continuous slice-by-slice comparison, the specific single slice demonstrating the maximum muscle belly thickness was definitively identified, and the boundaries were manually delineated while excluding the tendons (15).

(2) OFV assessment. OFV was quantified on 3D T1-weighted fat-phase images using multiplanar reconstruction to delineate orbital fat boundaries. The initial coronal slice was defined at the level of the maximum eyeball/lens diameter, and the final slice was located near the orbital apex where the anatomical structures became indistinct. Regions of interest were manually delineated while excluding the EOMs, optic nerve, lacrimal gland, and vasculature. Total volume (cm³) was calculated using Philips IntelliSpace Portal 12.0.

All measurements were performed in triplicate, and the mean values were used for analysis. Intra- and inter-observer reliability were confirmed with ICC values > 0.85. For OFV measurements, discrepancies > 10% were adjudicated by a third senior radiologist.

### Definition of therapeutic responses and classification of therapeutic outcomes

To characterize the heterogeneity of therapeutic responses and clinical outcomes, the 24 enrolled patients were categorized into five clinical phenotypes based on changes in inflammatory activity following one cycle of TCZ treatment. The definitions of these phenotypes are summarized in **Table 1**.

**Table 1:** Definition of the five clinical phenotypes for therapeutic response classification.

Phenotype	Core Definition	Key Criteria	Clinical Characteristics	Threshold Justification
Superior Response	Steroid-naive patients achieving rapid disease resolution and stable remission	CAS ≥ 3 at T1 and ≤ 1 at T2 and T3	Rapid resolution of inflammation Significant EOM regression Surgery avoided	CAS ≤ 1: EUGOGO inactive definition (5)
Sequential Therapy	TCZ as a pre-surgical bridging followed by surgery	CAS reduction ≤ 2 at T2 Persistent visual impairment required orbital decompression (T4)	Severe disease Inflammation controlled Structural issues require subsequent surgery	CAS ≤ 2: EUGOGO recommendation before decompression surgery (5)
Steroid-Refractory	Prior glucocorticoid failure but robust response to TCZ	CAS ≥ 3 after steroid course at T1 CAS ≤ 1 at T2 and T3	Steroid-resistant rescued by TCZ Significant CAS improvement	CAS reduction ≤ 2: EUGOGO criteria for corticosteroid resistance (5)
Primary Non-responder	Negligible clinical benefit from TCZ	CAS reduction ≤ 1 at T2 Remained CAS ≥ 3 at T3	Limited inflammatory control Minimal structural change Requires alternative therapy	CAS ≥ 3 at T3: EUGOGO active definition (5)
Clinical Relapse	Initial response followed by inflammatory rebound	Rapid inflammatory resolution: CAS ≤ 1 at T2 Inflammation Relapse: CAS ≥ 3 at T3	Transient inflammatory remission IL-6 surge Requires extended treatment	CAS ≥ 3: EUGOGO active definition (5)

### Statistical analysis

Statistical analyses were performed using SPSS (v. 26.0) and GraphPad Prism (v. 10.0). Continuous variables were assessed for normality using the Shapiro-Wilk test and are presented as mean ± SD or median (IQR), as appropriate. Clinical outcomes at the three time points (T1, T2, and T3) were compared using repeated-measures ANOVA or the Friedman test, followed by Bonferroni *post hoc* correction. Radiographic parameters (EOM CSA) between T1 and T2 were compared using the Wilcoxon signed-rank test. All statistical tests were two-sided, and *P* < 0.05 was considered statistically significant. Given the exploratory nature of the study and the lack of prior quantitative MRI data, a formal sample size calculation was not performed. The sample size (*N* = 24) was determined by the number of eligible patients during the study period and was consistent with previous single-center studies (12, 13).

## Results

### Baseline characteristics

A total of 24 patients with active moderate-to-severe TAO were enrolled in this study (**Table 2**). The median age of the cohort was 50.50 years (IQR 38.00–58.00), and slightly more than half of the participants were female (13, 54.20%). The median disease duration was 11.50 months. At T1, all patients presented with active inflammation, with a median CAS of 3. According to the NOSPECS classification, 9 patients (37.5%) were grade 3, 13 patients (54.17%) were grade 4, and 2 patients (8.33%) were grade 6. No patients were classified as grade 5 in this cohort. Six of the 24 enrolled patients (25%) exhibited interocular asymmetry of varying severity. In these cases, data from the more severely affected eye were used for subsequent analyses. Twelve patients (50.00%) were current smokers at enrollment, while the remaining 12 patients (50.00%) had no history of smoking. Four patients (16.70%) had previously received steroid therapy but showed a suboptimal response. The median total clinical follow-up duration was 2.0 months (IQR 1.0-5.0), with the longest follow-up extending to 11.0 months (**Table 2**).

**Table 2:** Baseline demographics and clinical characteristics of patients with TAO (*N* = 24).

Characteristic	Value (*N* = 24)
Demographics	
Age (years)	50.50 (38.00–58.00)
Female	13 (54.20%)
Male	11 (45.80%)
Current Smokers	12 (50.00%)
Clinical history	
Duration of graves’ disease (months)	12.50 (7.50–18.75)
Duration of TAO (months)	11.50 (8.00–17.75)
Prior steroid therapy	4 (16.70%)
Prior orbital surgery	0 (0.00%)
Ophthalmic assessments	
CAS	3.00 (3.00–3.75)
NOSPECS grade	4.00 (3.00–4.00)
Proptosis (mm)	20.00 (19.13–21.00)
VA	0.80 (0.50–1.00)
Serological profile	
TRAb (IU/L)	4.95 (2.67–10.79)
TSH (mIU/L)	1.11 (0.02–4.31)
FT3 (pmol/L)	3.64 (3.04–5.73)
FT4 (pmol/L)	1.38 (1.04–2.94)
IL-6 (pg/mL)	1.50 (1.50–2.34)

Data are presented as *n* (%) for categorical variables and median (Q1, Q3) for continuous variables.

### Ophthalmic assessment

**Figure 2:**
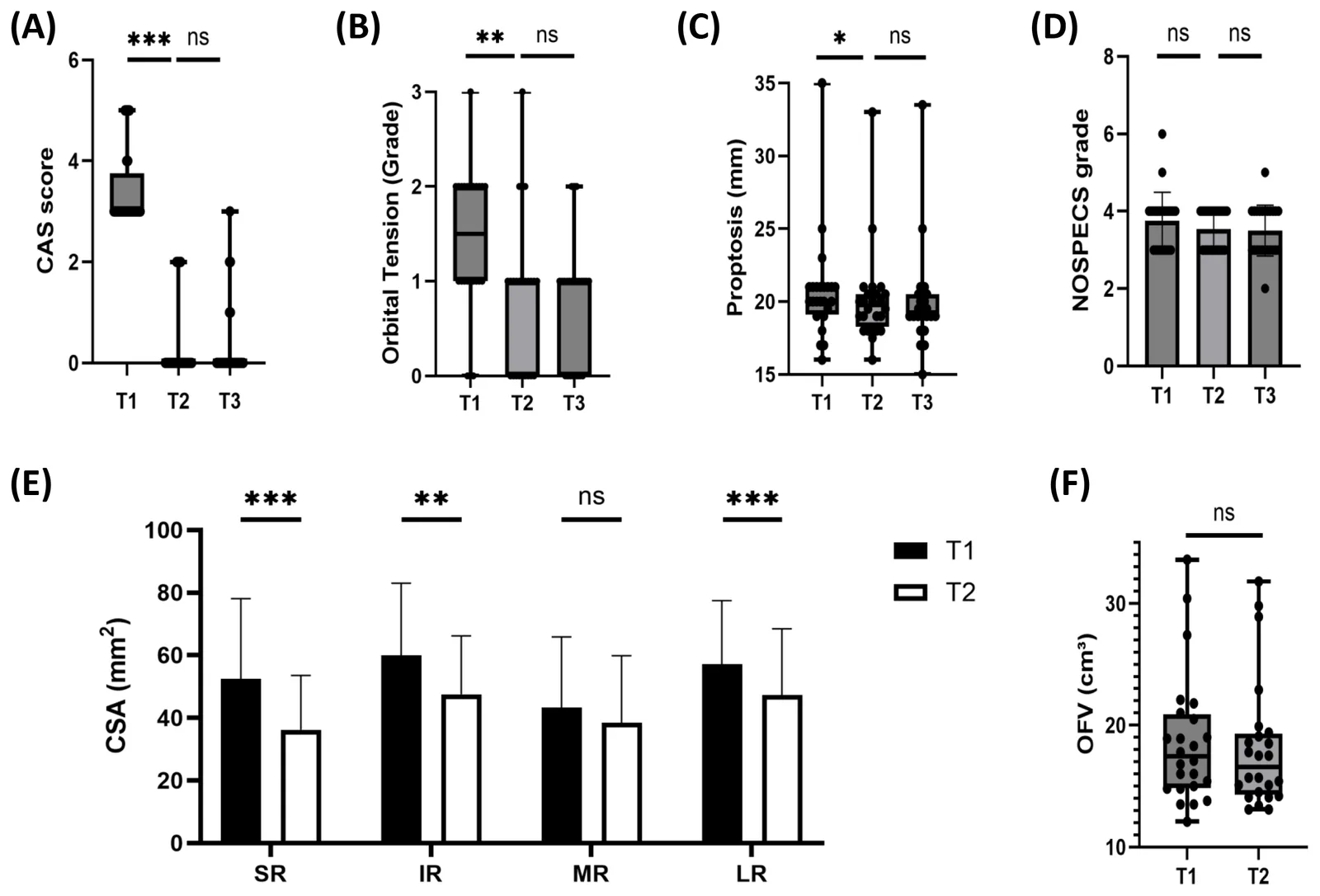
Changes in clinical outcomes and radiographic parameters following TCZ therapy. Serial assessments of CAS **(A)**, orbital tension grade **(B)**, and proptosis **(C)** across three time points (T1, T2, and T3). Improvements achieved at T2 were maintained at T3. **(D)** NOSPECS grade showing no statistically significant change across the three time points (*P* = 0.166). **(E)** Comparison of EOM CSA at T1 and T2. Significant reduction in volume was observed in the SR, IR, and LR (*P* < 0.05), whereas the MR (*P* > 0.05) showed structural stability. **(F)** Longitudinal changes in OFV between T1 and T2, showing no statistically significant reduction (*P* = 0.179). *P < 0.05, **P < 0.01, ***P < 0.001.

To evaluate primary therapeutic efficacy, a paired analysis (*N* = 24) was performed to compare clinical and radiographic parameters between T1 and T2. A comparison between T2 and T3 was used to assess the durability of the therapeutic response after treatment cessation (**Table 3**). Treatment with TCZ significantly reduced CAS. Specifically, CAS decreased from a median of 3.00 (IQR 3.00-3.75) at T1 to 0.00 (IQR 0.00-0.00) at T2 (*P* < 0.001), and remained stable at 0.00 (IQR 0.00-0.00) at T3 (**Figure 2A**). At T3, one patient (4.2%) had a CAS of 3. Low-level disease activity was observed in four additional patients: two (8.3%) had a CAS of 2, and two (8.3%) had a CAS of 1. The remaining 19 patients (79.2%) maintained a CAS of 0.

**Table 3:** Longitudinal changes in clinical ophthalmic and radiographic parameters from baseline to follow-up (*N* = 24).

Parameter	Baseline (T1)	Post-treatment (T2)	1-month follow-up (T3)	*P* value*^a^*	*P* value*^b^*	*P* value*^c^*
**CAS**	3.00 (3.00–3.75)	0.00 (0.00–0.00)	0.00 (0.00–0.75)	**< 0.001**	**< 0.001**	0.257
**Orbital Tension**	1.50 (1.00–2.00)	1.00 (0.00–1.00)	1.00 (0.00–1.00)	**0.002**	**< 0.001**	0.705
**Proptosis (mm)**	20.00 (19.13–21.00)	19.75 (18.25–20.50)	19.50 (19.00–20.50)	**0.020**	**0.033**	0.742
**NOSPECS Score**	4.00 (3.00–4.00)	4.00 (3. 00–4.00)	3.50 (3.00–4.00)	0.166	0.157	0.705
EOM CSA (mm^2^)						
SR	50.40 (31.10–71.95)	33.20 (22.33–47.88)	N/A	**< 0.001**	N/A	N/A
IR	54.90 (41.70–82.18)	46.20 (30.23–61.13)	N/A	**0.002**	N/A	N/A
LR	52.85 (45.20–63.70)	40.20 (34.30–57.18)	N/A	**< 0.001**	N/A	N/A
MR	35.00 (25.23–63.95)	28.95 (23.50–54.48)	N/A	0.170	N/A	N/A
**OFV (cm³)**	17.45 (14.85–20.88)	16.60 (14.28–19.33)	N/A	0.179	N/A	N/A

Data are presented as median (Q1, Q3). *P* < 0.05 was considered statistically significant. ^a^Comparison between T1 (Baseline) and T2 (Post-treatment) was performed using the Wilcoxon signed-rank test (*N* = 24). ^b^Comparison between T1 (Baseline) and T3 (1-month Follow-up) was performed using the Wilcoxon signed-rank test (*N* = 24). ^c^Comparison between T2 (Post-treatment) and T3 (1-month Follow-up) was performed using the Wilcoxon signed-rank test (*N* = 24).Bold values indicate statistical significance (P < 0.05).

Orbital tension grades showed a statistically significant reduction at T2 (*P* = 0.002) and remained stable at T3 (T2 vs. T3, *P* > 0.05) (**Figure 2B**). Hertel exophthalmometry demonstrated a slight but statistically significant reduction in proptosis, with median values decreasing from 20.00 mm (IQR 19.13-21.00) at T1 to 19.75 mm (IQR 18.25-20.50) at T2 (*P* = 0.020) (**Figure 2C**). This improvement was maintained at T3 (T2 vs. T3, *P* > 0.05). The total NOSPECS grade showed no statistically significant change (*P* = 0.166) (**Figure 2D**).

### Changes of EOM CSA and OFV

Quantitative orbital imaging demonstrated a significant reduction in EOM CSA following TCZ treatment (T1 vs. T2, **Table 3**).

Between T1 and T2, significant reductions in CSA were observed in the superior rectus (SR) (*P* < 0.001), inferior rectus (IR) (*P* = 0.002), and lateral rectus (LR) (*P* < 0.001). In contrast, the medial rectus (MR) showed no significant change in CSA (*P* = 0.170) (**Figure 2E**). Unlike the EOMs, OFV did not show a statistically significant reduction between T1 and T2 (*P* = 0.179) (**Figure 2F**).

### Serological, thyroid function, and inflammatory outcomes

**Figure 3:**
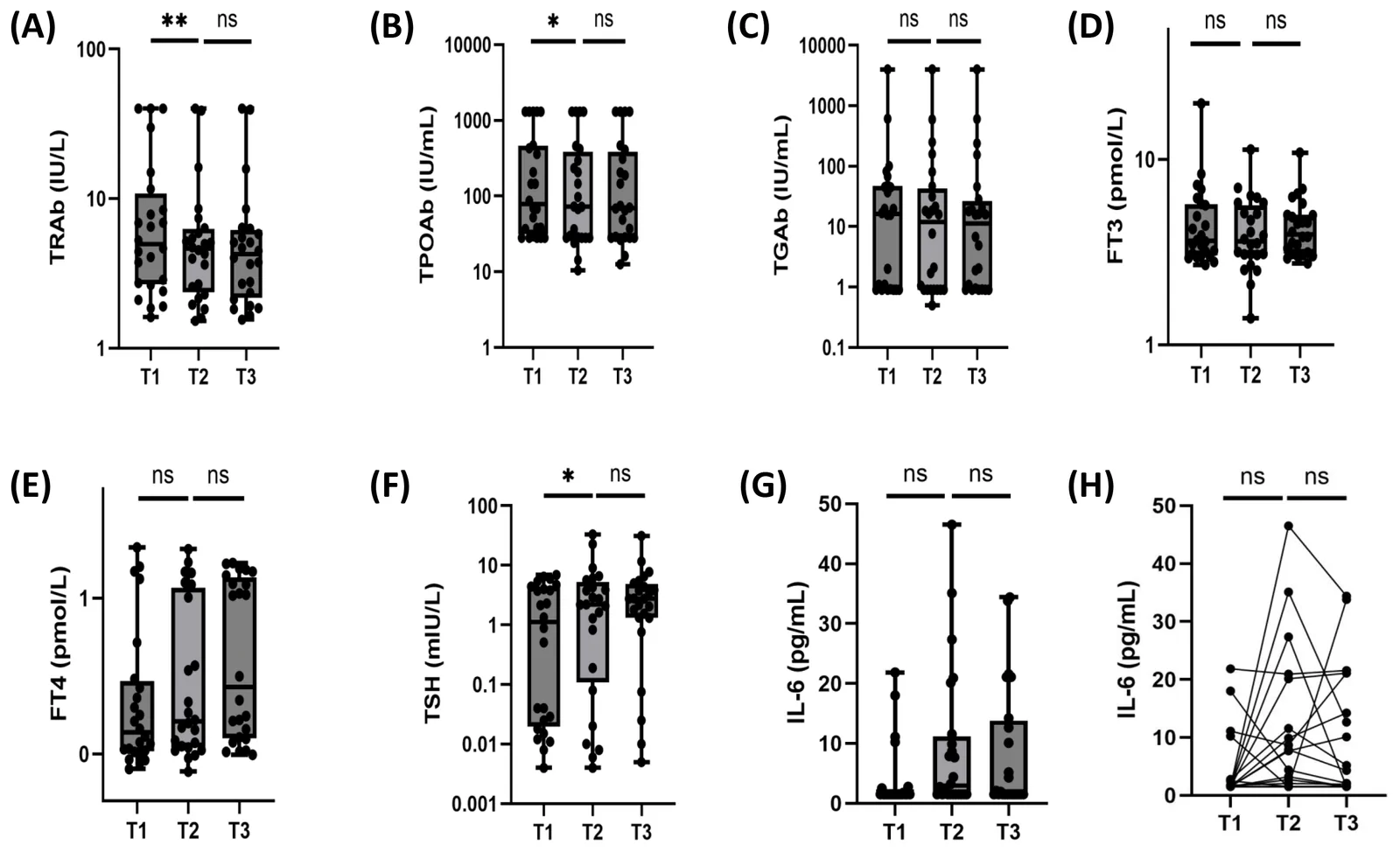
Longitudinal assessment of thyroid function and IL-6. Serial changes in serum TRAb **(A)**, TPOAb **(B)**, and TgAb **(C)** levels at T1, T2, and T3. Significant reductions were observed in TRAb and TPOAb from T1 to T2, whereas TgAb levels remained stable throughout the study period. Longitudinal changes in FT3 **(D)**, FT4 **(E)**, and TSH **(F)** levels. FT3 and FT4 levels remained stable within the reference range across all time points. TSH levels increased significantly from T1 to T2 (*P* = 0.015), while all values remained within the normal reference range (0.4-4.0 mIU/L). **(G)** Longitudinal assessment of serum IL-6 levels at T1, T2, and T3. **(H)** Individual patient trajectories of serum IL-6 levels across three time points, demonstrating inter-individual heterogeneity. *P < 0.05, **P < 0.01.

To evaluate systemic responses, serum autoantibodies and thyroid function were compared between T1 and T2. In addition, longitudinal changes in thyroid function parameters after TCZ discontinuation were assessed by comparing T2 and T3 (**Table 4**). Serum TRAb levels significantly decreased from a median of 4.95 IU/L at T1 to 4.62 IU/L at T2 (*P* = 0.007) (**Figure 3A**). TPOAb levels also showed a significant decline from T1 to T2 (*P* = 0.022) (**Figure 3B**), whereas TGAb levels remained stable over the same period (*P* = 0.936) (**Figure 3C**). Concurrently, thyroid function remained stable, with FT3 and FT4 levels within the reference range (*P* > 0.05) (**Figures 3D, E**). Although TSH levels increased significantly from 1.11 mIU/L at T1 to 2.19 mIU/L at T2 (*P* = 0.015) (**Figure 3F**), all values remained within the normal reference range of 0.4-4.0 mIU/L (21). Ultimately, comparisons between T2 and T3 revealed no significant differences in any thyroid parameters (*P* > 0.05).

**Table 4:** Changes in serological, inflammatory, and thyroid function parameters from baseline to follow-up (*N* = 24).

Parameter	Baseline (T1)	Post-treatment (T2)	1-month follow-up (T3)	*P* value*^a^*	*P* value*^b^*	*P* value*^c^*
Autoantibodies						
TRAb (IU/L)	4.95 (2.67–10.79)	4.62 (2.36–6.24)	4.25 (2.18–6.18)	**0.007**	**0.004**	0.964
TPOAb (IU/mL)	78.25 (31.33–459.50)	72.55 (28.00–386.00)	69.45 (28.13–384.25)	**0.022**	**0.031**	0.965
TGAb (IU/mL)	16.15 (0.91–47.18)	11.80 (0.90–42.78)	11.25 (0.92–26.43)	0.936	0.811	0.219
Inflammatory Markers						
IL-6 (pg/mL)	1.50 (1.50–2.34)	2.98 (1.50–11.15)	1.73 (1.50–13.83)	0.070	0.064	0.496
Thyroid Function						
TSH (mIU/L)	1.11 (0.02–4.31)	2.19 (0.10–5.30)	2.76 (1.32–4.85)	**0.015**	**0.006**	0.853
FT3 (pmol/L)	3.64 (3.04–5.73)	3.62 (3.06–5.65)	3.94 (3.11–5.01)	0.368	1.000	0.224
FT4 (pmol/L)	1.38 (1.04–2.94)	1.62 (1.11–11.68)	2.68 (1.26–13.54)	0.647	0.189	0.149

Data are presented as median (Q1, Q3). P < 0.05 was considered statistically significant. ^a^Comparison between T1 (Baseline) and T2 (Post-treatment) was performed using the Wilcoxon signed-rank test (*N* = 24). ^b^Comparison between T1 (Baseline) and T3 (1-month Follow-up) was performed using the Wilcoxon signed-rank test (*N* = 24). ^c^Comparison between T2 (Post-treatment) and T3 (1-month Follow-up) was performed using the Wilcoxon signed-rank test (*N* = 24).Bold values indicate statistical significance (P < 0.05).

Serum levels of IL-6, a systemic inflammatory mediator, were monitored throughout the study period. The median IL-6 level increased from 1.50 pg/mL at T1 to 2.98 pg/mL at T2 (*P* > 0.05), and subsequently decreased to 1.73 pg/mL at T3 (**Figure 3G**). Notably, IL-6 dynamics exhibited inter-individual heterogeneity (**Figure 3H**).

### Safety profile

Safety outcomes and hematological parameters were compared between T1 and T2, demonstrating that TCZ was well tolerated (**Table 5**). No severe infusion-related hypersensitivity reactions occurred during the treatment period, despite the absence of premedication. Hematological monitoring revealed no evidence of myelosuppression between T1 and T2. In addition, no statistically significant changes were observed in WBC counts (*P* = 0.898), NEUT counts (*P* = 0.932), LYM counts (*P* = 0.457), HGB (*P* = 0.474), or PLT (*P* = 0.484) between T1 and T2. Furthermore, no grade 3/4 neutropenia was observed at any time during the study period.

**Table 5:** Safety assessment: hematological and liver function parameters from baseline to follow-up (*N* = 24).

Parameter	Baseline (T1)	Post-treatment (T2)	1-month follow-up (T3)	*P* value*^a^*	*P* value*^b^*	*P* value*^c^*
Hematology						
WBC (10^9^/L)	5.69 (4.99–7.28)	6.05 (4.55–7.00)	5.68 (4.57–6.97)	0.898	0.830	0.568
NEUT Counts (10^9^/L)	3.23 (2.68–4.60)	3.37 (2.59–4.34)	3.07 (2.61–4.46)	0.932	0.830	0.324
NEUT Percentage (%)	59.55 (51.20–65.78)	59.85 (53.75–63.50)	58.80 (54.50–65.05)	0.855	0.867	0.989
LYM Counts (10^9^/L)	1.97 (1.58–2.33)	1.95 (1.50–2.29)	1.95 (1.58–2.34)	0.457	0.749	0.067
LYM Percentage (%)	32.45 (25.80–41.05)	31.90 (28.15–36.73)	33.80 (28.53–39.73)	0.915	0.867	0.775
HGB (g/L)	143.50 (135.75–157.50)	147.00 (137.50–156.50)	145.00 (139.00–155.75)	0.474	0.257	0.580
PLT (10^9^/L)	204.00 (191.25–223.25)	200.0 (171.50–233.00)	206.00(175.00–241.75)	0.484	0.484	0.338
Liver Function						
ALT (U/L)	23.95 (18.63–32.48)	22.75 (18.85–26.70)	24.90(18.20–31.95)	0.323	0.988	0.875
AST (U/L)	19.00 (15.63–24.20)	18.35 (15.48–21.00)	19.00 (16.63–24.08)	0.331	0.573	0.053
GGT (U/L)	27.80 (18.05–35.88)	24.65 (17.65–43.75)	25.70 (17.05–37.38)	0.742	0.772	0.775
ALP (U/L)	85.60 (65.15–131.45)	86.55 (63.93–131.78)	82.60 (65.10–128.63)	**0.049**	**0.011**	0.271
TBIL (μmol/L)	13.30 (10.75–16.33)	12.65 (11.30–19.33)	13.90 (11.83–16.18)	0.353	0.558	0.528
DBIL (μmol/L)	3.55 (2.70–4.70)	3.30 (2.45–4.58)	3.50 (2.73–4.30)	0.473	0.844	0.753
IBIL (μmol/L)	10.12 (7.43–11.85)	10.40 (7.98–14.90)	10.25 (8.80–12.50)	0.208	0.361	0.492

Data are presented as median (Q1, Q3). *P* < 0.05 was considered statistically significant. ^a^Comparison between T1 (Baseline) and T2 (Post-treatment) was performed using the Wilcoxon signed-rank test (*N* = 24). ^b^Comparison between T1 (Baseline) and T3 (1-month Follow-up) was performed using the Wilcoxon signed-rank test (*N* = 24). ^c^Comparison between T2 (Post-treatment) and T3 (1-month Follow-up) was performed using the Wilcoxon signed-rank test (*N* = 24).Bold values indicate statistical significance (P < 0.05).

Liver function tests indicated a favorable safety profile between T1 and T2. Levels of ALT (*P* = 0.323), AST (*P* = 0.331), GGT (*P* = 0.742), and TBIL (*P* = 0.353) remained stable, indicating no evidence of significant hepatotoxicity during the treatment period. Although ALP levels showed statistically significant fluctuations (*P* = 0.049 for T1 vs. T2), median values remained within the normal reference range throughout the study (21). In addition, longitudinal monitoring of hematological and hepatic parameters between T2 and T3 revealed no significant changes (*P* > 0.05) (**Table 5**). Considering potential concerns regarding hypercoagulability associated with biologic therapy, routine lower extremity venous ultrasonography was performed. No evidence of DVT or other thromboembolic complications was observed in any patient during the treatment course.

### Representative case presentations and response heterogeneity

**Figure 4:**
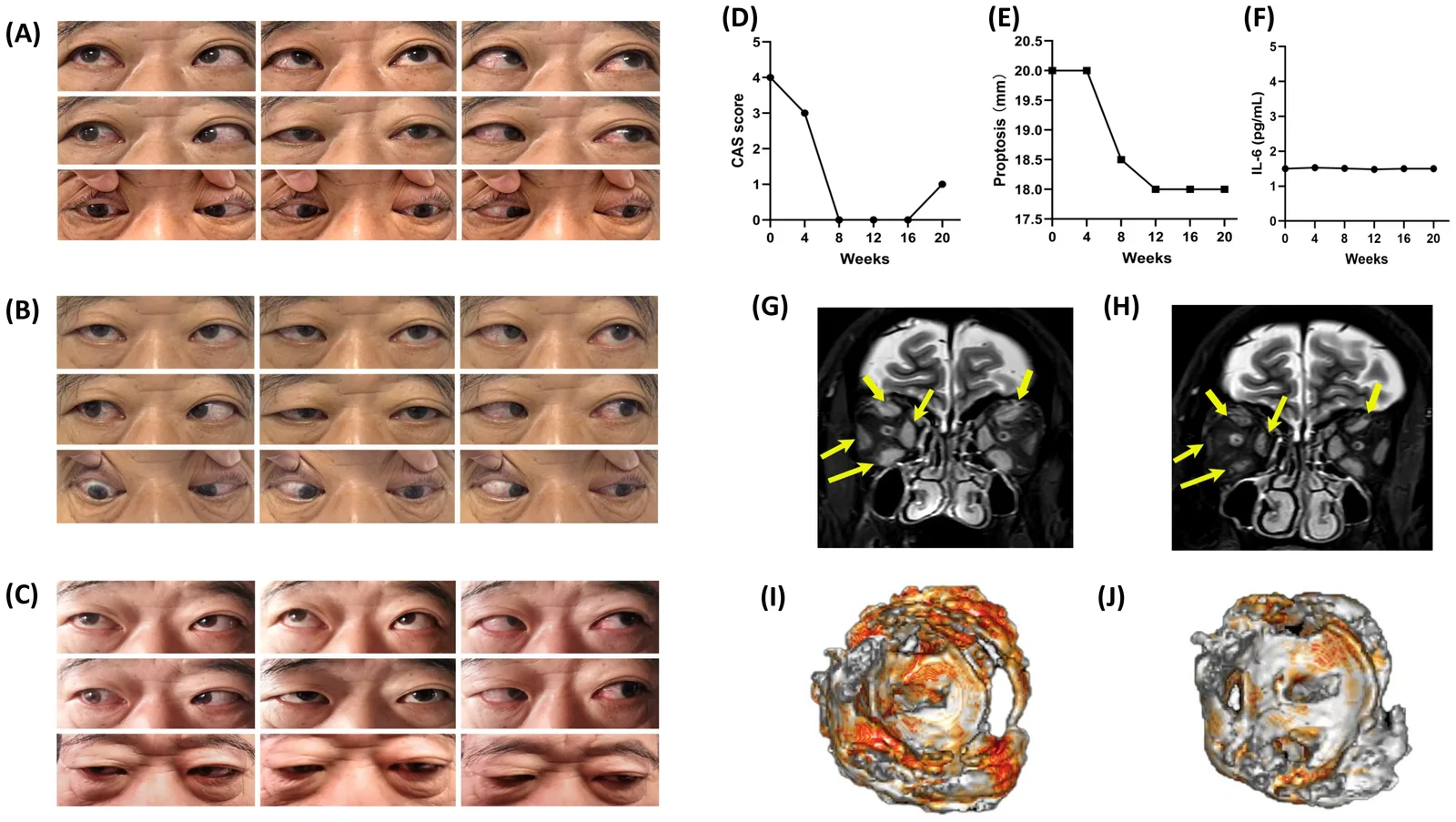
Clinical and radiographic trajectory of Case 1 (Superior Response). Serial nine-direction gaze photographs at T1 **(A)**, T2 **(B)**, and T3 **(C)**. Longitudinal changes in CAS **(D)**, proptosis (mm) **(E)**, and IL-6 (pg/mL) **(F)**, illustrating inflammatory inactivation over a 20-week period following TCZ therapy. Coronal short tau inversion recovery (STIR) MRI at T1 **(G)** and T2 **(H)**. Yellow arrows indicate marked reduction of inflammatory edema within the EOMs. Three-dimensional reconstructions demonstrated a marked reduction in EOM hypertrophy from T1 **(I)** to T2 **(J)**.

**Figure 5:**
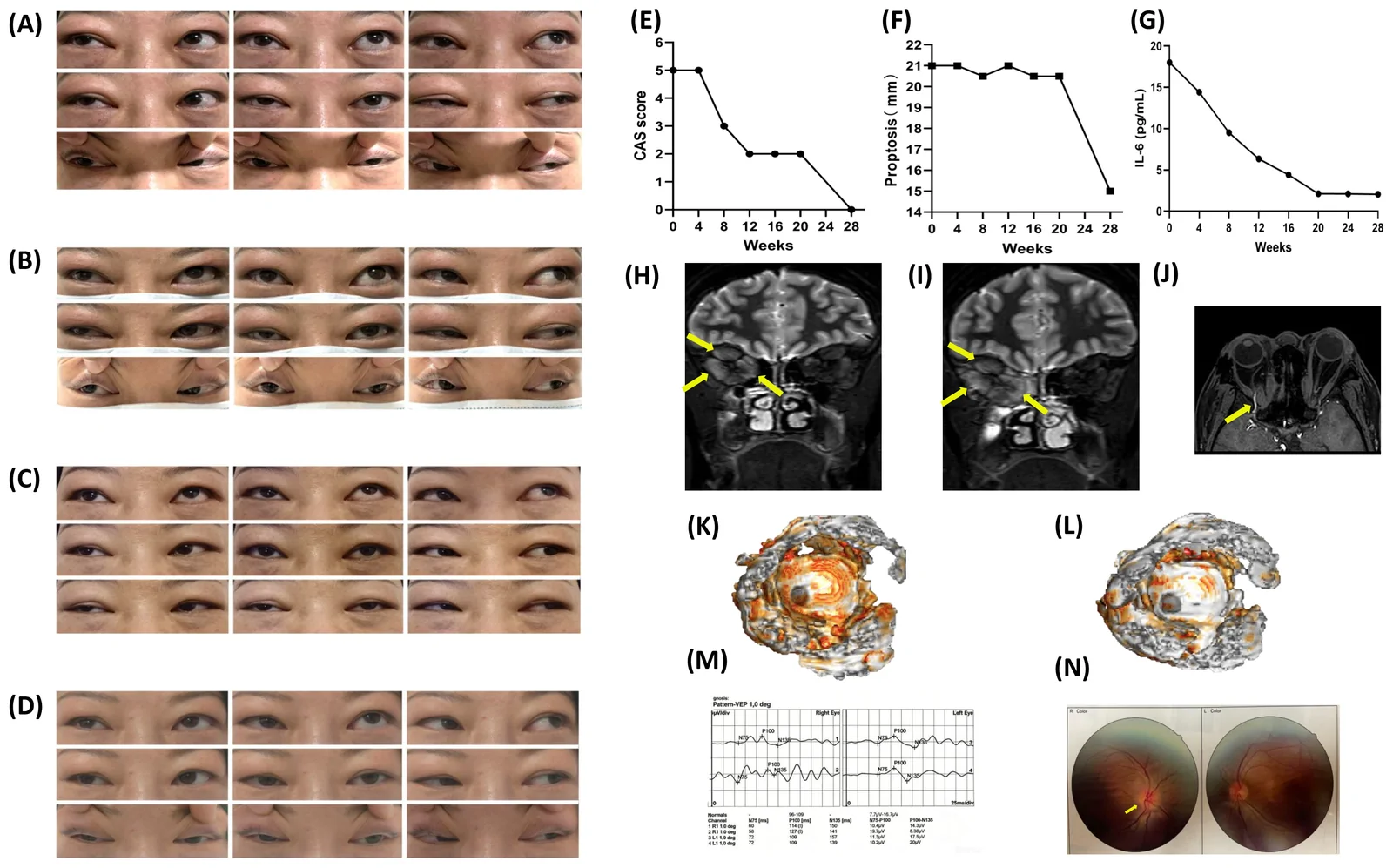
Clinical and radiographic trajectory of Case 2 (Sequential Therapy). Serial nine-direction gaze photographs at T1 **(A)**, T2 **(B)**, T3 **(C)**, and T4 **(D)**. Longitudinal changes in CAS **(E)**, proptosis (mm) **(F)**, and IL-6 (pg/mL) **(G)**, illustrating the synergistic effect of sequential therapy. TCZ therapy facilitated resolution of active inflammation, establishing a quiescent baseline for subsequent orbital decompression. Coronal STIR MRI at T1 **(H)** and T2 **(I)**. Imaging shows negligible changes in EOM CSA. **(J)** Axial orbital MRI demonstrating a markedly engorged right superior ophthalmic vein. Three-dimensional orbital soft tissue reconstructions demonstrating structural changes from T1 **(K)** to T2 **(L)**. **(M)** Pattern visual evoked potential showing prolonged P100 latency in the right eye. **(N)** Fundus photograph showing optic disc edema in the right eye.

To delineate the diverse clinical phenotypes of TAO and their corresponding therapeutic responses, five representative cases were ultimately selected. Among the 24 enrolled patients, 15 patients (62.50%) exhibited the Superior Response phenotype, 2 patients (8.3%) exhibited the Sequential Therapy phenotype, 4 patients (16.67%) exhibited the Steroid-Refractory phenotype, 2 patients (8.33%) exhibited the Primary Non-responder phenotype, and 1 patient (4.17%) exhibited the Clinical Relapse phenotype. We detailed two representative cases (the Superior Response and Sequential Therapy phenotypes) below (**Table 6**). Brief summaries of the remaining three phenotypes (Steroid-Refractory, Primary Non-responder and Clinical Relapse) are provided at the end of this section, with their detailed clinical and radiographic trajectories available in the **Supplementary Material** (**Supplementary Figure Legends**; **Supplementary Table 1**; **Supplementary Figures 1**-**3**). Safety assessments revealed no significant abnormalities in any of the cases, and nine-direction gaze photographs were consistent with clinical findings across all cases (**Figures 4A–C**, **5A**—**D**; **Supplementary Figures 1A–C**, **2A**—**C**, **3A**-**C**).

**Table 6:** Longitudinal profiles of clinical, serological, and radiographic parameters in two representative therapeutic response scenarios.

Parameters	CASE1 (Superior Responder)			CASE2 (Sequential Therapy)			
Clinical features and Phenotype	Rapid inactivation, profound EOM regression, and stable remission			Successful clinical inactivation with TCZ, followed by orbital decompression to resolve persistent proptosis			
Time Point	T1	T2	T3	T1	T2	T3	T4*^a^*
CAS	4	0	1	5	2	2	0
Proptosis (mm)	20.00	18.00	18.00	21.00	20.50	20.50	15.00
IL-6 (pg/mL)	1.50	1.50	1.50	18.01	4.41	2.12	2.06
Orbital Tension	2	2	1	3	3	2	0
SR (mm2)	85.90	38.30	N/A	50.70	60.10	N/A	N/A
IR (mm2)	42.60	39.50	N/A	45.30	71.40	N/A	N/A
LR (mm2)	73.10	59.80	N/A	73.30	78.80	N/A	N/A
MR (mm2)	30.50	61.70	N/A	67.90	106.20	N/A	N/A
OFV (cm³)	18.90	17.80	N/A	13.50	13.40	N/A	N/A
TRAb (IU/L)	7.85	7.40	6.30	2.10	2.15	2.12	2.09
FT3 (pmol/L)	5.65	5.83	3.82	6.89	6.35	6.52	6.50
FT4 (pmol/L)	21.15	20.60	13.94	2.29	2.15	2.21	2.20
TSH (mIU/L)	0.02	0.01	5.01	0.00	0.01	0.01	0.01

^a^In Case 2, T4 denotes the observation point following orbital decompression surgery, which was performed two months after T3. Orbital tension was graded as 0 (Tn), 1 (T + 1), 2 (T + 2), or 3 (T + 3).

Case 1 (Superior Responder), a previously untreated patient with active TAO, exhibited an excellent response to TCZ treatment. CAS decreased from 4 at T1 to 0 at T2 and remained at 1 at T3 (**Figure 4D**). Proptosis decreased from 20 mm to 18 mm at T2 and remained stable at T3 (**Figure 4E**). Orbital tension improved from 2 at T1 to 1 at T3, while IL-6 remained at 1.5 pg/mL throughout (**Figure 4F**). MRI confirmed marked changes in extraocular muscles (**Table 6**), with SR decreasing from 85.90 (**Figure 4G**) to 38.30 mm^2^ (**Figure 4H**) and IR decreasing from 42.60 (**Figure 4G**) to 39.50 mm^2^ (**Figure 4H**). Thyroid function stabilized at T3, with TSH recovering to 5.01 mIU/L. Three-dimensional reconstructions demonstrated a marked reduction in EOM hypertrophy from T1 (**Figure 4I**) to T2 (**Figure 4J**).

Case 2 (Sequential Therapy) illustrates complete sequential progression from high inflammatory activity to definitive surgical rehabilitation. CAS decreased from 5 at T1 to 2 at T2, remained at 2 at T3, and decreased to 0 at T4 (the observation point following orbital decompression surgery, which was performed two months after T3) (**Figure 5E**). Proptosis showed a slight reduction from 21.00 mm at T1 to 20.50 mm at T2 and T3, followed by a marked reduction to 15.00 mm at T4 (**Figure 5F**). Orbital tension improved from 3 at T1/T2 to 2 at T3, and further to 0 at T4. IL-6 decreased from 18.01 pg/mL at T1 to 4.41 pg/mL at T2 and remained stable thereafter (**Figure 5G**). MRI showed persistent EOM enlargement without significant volumetric reduction between T1 (**Figure 5H**) and T2 (**Figure 5I**). Orbital MRI demonstrated EOM hypertrophy, apical crowding, optic nerve compression, and marked engorgement of the right superior ophthalmic vein (**Figure 5J**). Thyroid function remained in a hyperthyroid state with suppressed TSH (< 0.01 mIU/L) and elevated TRAb (increased from 2.10 to 2.15 IU/L) (**Table 6**). Three-dimensional reconstructions demonstrated a visible reduction in active inflammatory signals from T1 (**Figure 5K**) to T2 (**Figure 5L**), but persistent structural crowding and minimal changes in OFV (13.50 (**Figure 5K**) to 13.40 cm³ (**Figure 5L**)) ultimately necessitated surgical decompression. At T2, visual acuity showed a marked interocular discrepancy (right 0.2 vs left 1.0). P-VEP showed prolonged P100 latency in the right eye (114 ms and 127 ms) (**Figure 5M**), and fundus photography revealed optic disc edema (**Figure 5N**). Right orbital decompression was performed, reducing proptosis to 15.00 mm and improving visual acuity to 0.8. Left orbital decompression was performed one month later.

Meanwhile, **Supplementary Case 1** (Steroid-Refractory), a patient with prior high-dose intravenous glucocorticoid failure, exhibited a marked clinical and structural response to TCZ. **Supplementary Case 2** (Primary Non-responder) demonstrated limited clinical benefit and minimal EOM regression from TCZ. Lastly, **Supplementary Case 3** (Clinical Relapse) exhibited a transient therapeutic response followed by a post-treatment relapse and IL-6 surge.

## Discussion

### Clinical efficacy of TCZ and its mechanisms

This retrospective study demonstrates that TCZ induces a significant therapeutic response and favorable clinical outcomes in patients with TAO. Following a single treatment cycle, CAS rapidly decreased to a non-active state (CAS ≤ 1), indicating prompt resolution of acute inflammation. These findings are consistent with the randomized controlled trial conducted by Perez-Moreiras et al. (11), which reported significant efficacy of TCZ in reducing CAS.

Our findings are also in agreement with previous reports suggesting that TCZ achieves comparable CAS reductions in both steroid-naive and steroid-resistant patients (22). This consistency across subgroups highlights the broad anti-inflammatory effects of TCZ. Clinically, its efficacy as a first-line agent appears comparable to its performance in steroid-resistant cases, suggesting that TCZ may serve as a viable alternative or primary treatment option in selected treatment-naive patients with systemic inflammatory activity.

The marked reduction in orbital pressure, accompanied by improvement in local signs, indicates effective alleviation of intraorbital congestion. This synchronous improvement is primarily attributed to targeted inhibition of the IL-6 signaling pathway by TCZ. This mechanism shifts the clinical focus from subjective inflammatory resolution to a measurable reduction in orbital tissue pressure. In the acute phase of TAO, IL-6 binds to receptors on orbital fibroblasts (OFs), activating downstream signaling pathways that upregulate hyaluronic acid synthase. This process leads to excessive glycosaminoglycan production, contributing to localized osmotic edema. TCZ competitively inhibits IL-6 receptor binding, thereby interrupting this pathological cascade. This blockade reduces interstitial fluid accumulation and promotes resorption of inflammatory exudates, resulting in decreased orbital pressure. Case 1 exemplifies these strong anti-inflammatory effects, as the patient was classified as a high responder within the cohort. Clinical manifestations included a rapid decline in CAS, reduction in orbital pressure, and prompt relief of subjective symptoms such as orbital pain.

Although the reduction in proptosis from 20.00 mm to 19.75 mm reached statistical significance (*P* = 0.020), the absolute change was only 0.25 mm, which is clinically minimal and falls below the inherent measurement error of Hertel exophthalmometry under routine conditions. This numerical variation does not indicate a limitation of treatment efficacy but rather suggests that proptosis alone may underestimate the true therapeutic effect. In contrast, changes in EOM CSA were more pronounced and objectively measurable. These imaging findings indicate that TCZ achieves a form of drug-induced decompression by reducing interstitial edema within hypertrophic muscles. However, due to the anatomical constraints of the bony orbit, regression of muscle edema may not be fully reflected in changes in exophthalmos. Therefore, our results suggest that minor changes in proptosis should not be used as the primary criterion for evaluating TCZ efficacy. Clinical assessment should instead prioritize its ability to suppress active orbital inflammation and prevent compressive optic neuropathy.

### Heterogeneity of EOM regression and the changes of OFV

The selective EOM responsiveness to TCZ observed in this study, particularly the contrast between SR and MR, warrants further mechanistic exploration at the cellular and molecular levels. The primary pathological basis of diffuse EOM hypertrophy in TAO is excessive synthesis of highly hydrophilic glycosaminoglycans (GAGs) (1, 23). This includes hyaluronic acid (HA), which is produced by orbital fibroblasts in response to local IL-6 stimulation. Traditional glucocorticoids exert their effects through non-specific, broad immunosuppression, however, they frequently show limited efficacy in suppressing established IL-6 signaling loops. This limitation may account for treatment resistance observed in certain patients, such as **Supplementary Case 1**. TCZ binds competitively and with high specificity to the IL-6 receptor (IL-6R), effectively interrupting downstream Janus kinase/signal transducer and activator of transcription (JAK/STAT) signaling and suppressing HA synthesis at its origin (24). As HA degrades and interstitial fluid is reabsorbed, a measurable anatomical regression of the EOMs occurs. **Supplementary Case 1**, which did not respond to initial high-dose glucocorticoid pulse therapy, subsequently received TCZ. After this switch, TCZ treatment resulted in a substantial reduction in muscle volume. This radiological finding suggests that TCZ may induce a form of “pharmacological decompression”, effectively addressing persistent inflammation in steroid-resistant cases.

Interestingly, this MRI-based anatomical response demonstrated marked differential muscle responsiveness. Specifically, the MR did not show a statistically significant volume change, whereas the SR exhibited the most pronounced reduction among all EOMs. The MR is typically the first muscle affected in the course of TAO and may therefore have already undergone chronic structural remodeling or fibrosis-associated changes, in which TCZ exhibits limited anti-inflammatory efficacy (25, 26).

While direct histological confirmation was lacking in this retrospective cohort, our interpretation is supported by converging indirect evidence, including the early and preferential involvement of MR in TAO, its differential responsiveness relative to other EOMs, and previous T1 mapping studies demonstrating collagen-driven extracellular expansion in inactive disease (27). Accordingly, this mechanism should be viewed as a well-supported hypothesis rather than a definitive finding, and future studies incorporating histological or fibrosis-specific imaging validation are warranted.

Two complementary mechanisms may account for this differential therapeutic response. First, in terms of inflammatory chronicity, EOMs involved early in the disease course, such as the MR and IR, may undergo phenotypic transformation of orbital fibroblasts under prolonged chronic inflammation. Histopathological and quantitative T1 mapping studies have shown that the pathological profile of EOMs shifts from acute inflammation characterized by glycosaminoglycan accumulation to chronic remodeling dominated by irreversible collagen deposition (27). At this stage, tissue composition is dominated by fibrosis-associated remodeling rather than active interstitial edema, leaving limited target substrate for TCZ, which results in reduced anatomical responsiveness to biologic therapy. Second, this heterogeneous response reflects intrinsic biological variability within the orbital microenvironment, in which different EOMs exist at asynchronous pathological stages. In this overlapping state of active inflammation and fibrosis-associated changes, certain muscles (such as the MR) may have already progressed to irreversible fibrosis-associated degeneration, whereas others (such as the SR) remain in the active inflammatory phase. Specifically, the SR represents an actively inflamed state characterized by high IL-6 receptor expression and prominent interstitial edema, making it highly responsive to IL-6R blockade (11). Conversely, the MR, affected earlier in the disease course, may have typically progressed beyond this optimal therapeutic window. This reduced pharmacological responsiveness likely results from gradual downregulation of IL-6 receptor expression as the muscle potentially transitions into a TGF-β-mediated fibrosis-associated state. Overall, this difference underscores that the anatomical efficacy of TCZ is determined by the interaction between systemic drug activity and muscle-specific pathological chronicity.

Our imaging analysis showed that OFV remained relatively stable before and after treatment, suggesting that TCZ primarily targets muscle edema rather than adipose tissue expansion. Koumas et al. described phenotypic heterogeneity in orbital fibroblasts, and our clinical observations support these findings (28). Thy-1^+^ orbital fibroblasts are predominantly located within EOM compartments. IL-6 signaling activates these cells to secrete HA, leading to localized edema. In contrast, most orbital fibroblasts in orbital adipose tissue are Thy-1^-^ cells, which exhibit adipogenic potential driven by IGF-1R and PPAR-γ pathways, contributing to significant OFV expansion (1, 3). TCZ specifically targets IL-6R and interrupts the pro-edema signaling cascade in Thy-1^+^ cells, while not directly inhibiting adipogenic differentiation. This mechanism provides a plausible explanation for the absence of significant OFV reduction observed in this cohort, although the limited sample size warrants cautious interpretation of this negative finding (29).

### Immune homeostasis regulation

Beyond local effects, TCZ also exhibited systemic immunomodulatory effects in this cohort. A significant decrease in pathogenic TRAb levels was observed, accompanied by a statistically significant increase in TSH, which may indicate partial reconstitution of the hypothalamic-pituitary–thyroid (HPT) axis. From an endocrine perspective, Smith et al. highlighted the importance of reduced TRAb titers, as this alleviates abnormal stimulation of TSH receptors on thyroid follicular cells and thereby reduces excessive thyroid hormone release (2, 30). The resulting reduction in peripheral thyroid hormone burden weakens negative feedback on the anterior pituitary, allowing suppressed TSH secretion to recover (1). Although TSH change reached statistical significance between T1 and T2 (*P* = 0.015), median TSH levels remained within the physiological reference range across all time points (T1, T2, and T3) (0.4-4.0 mIU/L) (21). Therefore, this fluctuation is interpreted as a restoration of systemic endocrine homeostasis rather than TCZ-induced thyroid dysfunction. These serological findings support the role of TCZ in restoring HPT axis homeostasis through inhibition of systemic immune cascades. From an immunological perspective, as described by Tanaka et al. (24), IL-6 is a key driver of B-lymphocyte differentiation into plasma cells and subsequent autoantibody production. By blocking IL-6R, TCZ interrupts this humoral immune amplification pathway and suppresses the generation of autoreactive plasma cells, leading to a reduction in serum TRAb titers (13, 24). This effect is consistent with findings from previous trials, including the randomized controlled trial conducted by Perez-Moreiras et al. (11). These data suggest that the primary mechanism of TCZ is the inhibition of downstream effector signaling in target tissues rather than direct elimination of upstream pathogenic autoantibodies. Although autoantibodies remain present in circulation, targeted blockade of IL-6R in affected tissues effectively disrupts the inflammatory amplification loop. It should be emphasized that serum TRAb represents a relatively delayed biomarker for evaluating TCZ efficacy and should be interpreted in conjunction with CAS, proptosis, orbital tension, and orbital imaging findings. While changes in TRAb and TSH reflect systemic immunomodulatory effects of TCZ, the concurrent elevation of serum IL-6 observed in the same patients requires a distinct pharmacodynamic interpretation, as discussed below.

### The “IL-6 paradox”

The feedback-associated elevation of serum IL-6 observed at T2 is consistent with the classic “IL-6 Paradox” (2). However, rather than simply restating this phenomenon, a more detailed pharmacodynamic interpretation is required to clarify its clinical significance. Under physiological conditions, circulating IL-6 is primarily cleared via receptor-mediated endocytosis. TCZ is a competitive IL-6 receptor antagonist that binds with high affinity to both membrane-bound and soluble IL-6 receptors. By saturating these receptors, TCZ effectively blocks the main clearance pathway for free IL-6, resulting in transient accumulation of IL-6 in the circulation. This laboratory finding should not be misinterpreted as systemic inflammatory exacerbation. In contrast, within our cohort, except for treatment-resistant patients such as **Supplementary Case 2**, who showed persistently elevated serum IL-6 levels across all time points (T1, T2, and T3), the remaining patients who exhibited a mild increase in serum IL-6 at T2 consistently demonstrated significant clinical improvement, as evidenced by marked reductions in both CAS and orbital tension. No patient developed worsening systemic inflammatory symptoms, nor were any new adverse events attributable to IL-6 accumulation observed. However, the current study did not measure soluble IL-6 receptor levels, TCZ serum concentrations, or pharmacodynamic markers of IL-6 pathway inhibition. Consequently, the observed IL-6 elevation is consistent with, but does not definitively prove, effective receptor blockade at the molecular level. Nevertheless, from a clinical standpoint, this limitation does not undermine the practical utility of the finding. The clinical-serological dissociation, patients with elevated IL-6 at T2 showed marked clinical improvement with no evidence of systemic inflammatory worsening, provides indirect but compelling evidence that the elevated IL-6 reflects target engagement rather than treatment failure. While prospective studies incorporating pharmacodynamic measurements are needed to confirm the exact mechanism, the current data are sufficient to reassure clinicians that IL-6 elevation following TCZ should not be misinterpreted as a sign of disease exacerbation.

This same pharmacokinetic mechanism may also explain the susceptibility to relapse in a subset of high-risk patients, as illustrated by **Supplementary Case 3**. In this patient, after completion of the standard 4-dose regimen, serum IL-6 rebounded markedly to 21.10 pg/mL at T3, accompanied by worsening proptosis. The pharmacodynamic trajectory is instructive. As TCZ is gradually eliminated, previously occupied IL-6 receptors become available again (2). At this stage, free IL-6, which had accumulated during treatment due to impaired clearance, binds to these newly available receptors. This rebound receptor engagement reactivates the JAK/STAT signaling pathway in orbital fibroblasts, reinitiating hyaluronic acid synthesis and inflammatory edema. However, this post-treatment IL-6 surge was not observed in patients who maintained stable therapeutic responses. For patients exhibiting post-treatment IL-6 rebound and subsequent relapse, extension of TCZ maintenance therapy beyond the conventional short-course regimen may be necessary. As demonstrated by Nishimoto et al. in the STREAM extension study (30), long-term TCZ maintenance achieves durable control of autoimmune disease activity. Although international protocols commonly recommend a fixed 4-dose regimen for TCZ therapy, extension of the treatment course should be considered in high-risk patients prone to relapse, rather than adhering rigidly to a fixed schedule.

### Preoperative application of TCZ for severe TAO

Another important clinical finding of this study is the potential application of TCZ as a preoperative strategy to optimize the orbital environment. In patients with marked apical crowding, such as Case 2, severe hypertrophy of the EOMs often limits the extent of decompression achievable with medical therapy alone. In these cases, the value of TCZ lies in its ability to create a more favorable local environment for subsequent surgical intervention by reducing acute inflammation, relieving congestion, and lowering orbital pressure. From a hemodynamic perspective, edematous EOMs contribute to a high-pressure state within the closed orbital space, resembling orbital compartment syndrome. This condition mechanically compresses the venous system, leading to increased tissue congestion and fragility. TCZ promotes the resolution of interstitial fluid, thereby reducing edema and alleviating vascular congestion. Consequently, this intervention may reduce the risk of intraoperative bleeding and serious complications, such as ischemic optic nerve injury, during subsequent bony decompression (11). Case 2 exemplifies this therapeutic strategy, as TCZ treatment initially achieved disease stabilization, allowing the patient to subsequently undergo orbital decompression. This combined approach reduced proptosis from 21.00 mm to 15.00 mm. A two-stage sequential strategy optimizes the management of severe TAO, in which targeted biologic therapy first suppresses active inflammation, followed by surgical decompression to correct residual anatomical distortion.

### Safety analysis

Safety data from this cohort confirm that TCZ was consistently well tolerated. This safety profile appears more favorable than that of traditional high-dose glucocorticoid pulse therapy. Although mild elevation of serum ALP was observed in a small subset of patients, this was interpreted as a transient adaptive hepatic response to the metabolism of large-molecule biologic agents (13). Moreover, these biochemical changes did not exceed clinical safety thresholds and required no intervention. In contrast to the multi-system adverse effects frequently associated with GCs (3), the adverse event profile of TCZ was comparatively limited and manageable (31). Importantly, with the exception of the relapse observed in **Supplementary Case 3**, no typical “withdrawal rebound” phenomenon was observed during follow-up among the remaining patients, whereas this phenomenon is commonly reported during steroid tapering. This stability underscores the potential advantage of TCZ in maintaining physiological equilibrium and achieving stable disease control.

### Clinical implications

**Figure 6:**
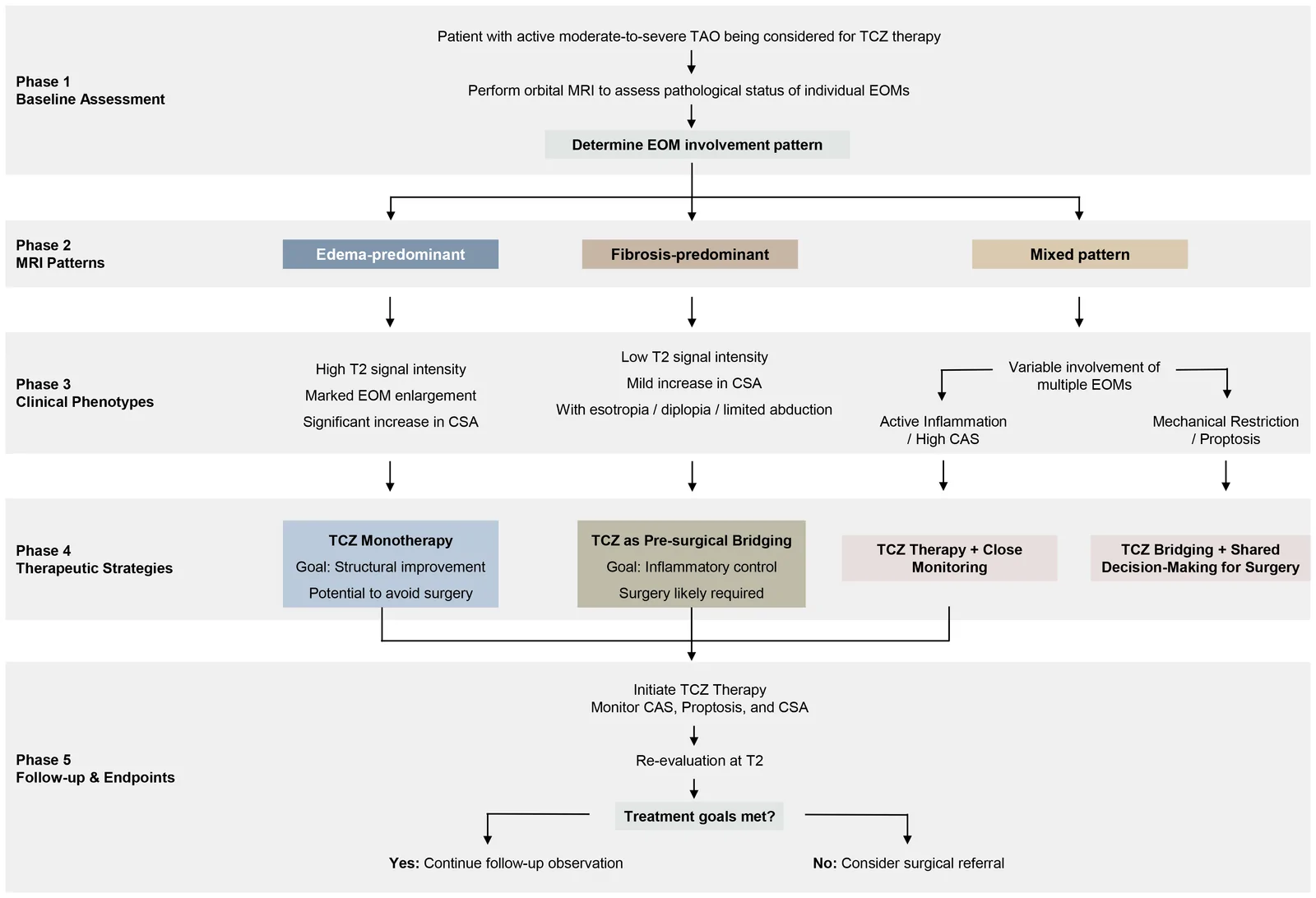
The framework for TCZ in active TAO. This flowchart delineates a practical approach to personalized treatment based on baseline EOM characteristics. Patients are stratified into edema-predominant, fibrosis-predominant, or mixed patterns using orbital MRI. These distinct phenotypes subsequently guide tailored therapeutic strategies, such as TCZ monotherapy and pre-surgical bridging, and inform long-term endpoint evaluation and surgical referral decisions.

The differential EOM responsiveness observed in this study may have implications for clinical decision-making. Based on these findings, we propose a practical framework for selecting patients for TCZ therapy and determining treatment goals according to baseline MRI characteristics (**Figure 6**).

In patients with MRI findings of increased T2 signal intensity and markedly enlarged EOM CSA, the underlying pathology is likely dominated by reversible inflammatory edema. In such cases, early initiation of TCZ may significantly reduce EOM CSA, achieve structural improvement, and potentially reduce the need for subsequent surgical intervention. This is exemplified by Case 1, which achieved stable remission without surgery. These patients may be considered the optimal candidates for TCZ monotherapy.

Conversely, in patients with MRI findings of low T2 signal intensity and minimal CSA enlargement, accompanied by clinical presentations such as esotropia, diplopia, or restricted abduction, fibrosis-associated changes are likely already present. In such cases, TCZ may effectively control active inflammation but may not fully resolve mechanical restriction. The therapeutic goal of TCZ in this setting should be positioned as a pre-surgical bridge to optimize orbital conditions and reduce intraoperative bleeding risk. The orbital decompression or strabismus surgery may eventually be required. Case 2 and **Supplementary Case 2** in our cohort exemplify this scenario, where the structural benefit of TCZ was limited, and both patients ultimately required surgical intervention.

For patients with mixed MRI patterns, treatment decisions should be guided by the predominant clinical concern. When inflammation and high CAS are the primary issues, TCZ is a reasonable choice, and the response should be closely monitored. When mechanical restriction or proptosis dominates, the patient and clinician should discuss the likelihood that surgery may ultimately be needed. In such cases, TCZ can serve as a bridge to optimize conditions before surgery.

This MRI-based phenotyping approach provides a practical imaging-guided framework for individualized treatment planning with TCZ. Patients with edema-predominant EOM involvement may be treated with TCZ monotherapy with the goal of avoiding surgery, whereas patients with fibrosis-predominant involvement should be informed that the primary value of TCZ lies in inflammation control and preoperative optimization. However, the proposed treatment algorithm (**Figure 6**) has not been prospectively validated. Its clinical utility and generalizability require confirmation in independent, larger-scale prospective studies in the future.

### Study limitations

This study provides clinically relevant insights, however, several limitations should be acknowledged. First, the small sample size (N = 24) and the single-arm, open-label design represent important methodological constraints. Given the potentially self-limiting nature of TAO, the absence of a parallel control group makes it difficult to reliably isolate the net therapeutic effect of TCZ. Nevertheless, the primary aim of this study was to generate hypotheses regarding the differential structural responses of individual EOMs to TCZ rather than to establish definitive comparative efficacy. Despite the limited sample size, the statistically significant reductions in the SR, IR, and LR CSAs provide evidence that the study was adequately powered to detect the main structural changes. Importantly, the interpretation of the negative findings for the MR and OFV warrants differentiation from the significant changes observed in other EOMs. The MR not only failed to show regression, but in several cases exhibited paradoxical enlargement, a directional pattern consistent with fibrosis-associated remodeling rather than reversible edema. This suggests that the lack of MR response is more likely a true biological negative reflecting chronic structural changes, rather than a false negative due to insufficient sample size. Similarly, the stability of OFV aligns with TCZ’s mechanism, which targets IL-6-driven muscle edema with minimal effect on orbital fat expansion. Although these mechanistic interpretations are speculative and require validation in larger cohorts, they are consistent with the established understanding of TCZ’s pharmacological specificity.

Second, radiological assessments were limited to T1 and T2, and routine MRI was not performed at T3. Consequently, long-term persistence of EOM regression after treatment discontinuation could not be radiologically confirmed, nor could subclinical recurrence be excluded. Furthermore, although previous studies have demonstrated that CSA serves as an effective and valid surrogate for volumetric measurement, future investigations incorporating complete 3D volumetric analysis are still needed to fully corroborate these anatomical findings. Third, the current study lacked fibrosis-related imaging biomarkers or histological validation. Consequently, the limited responsiveness observed in the MR remains a hypothesis rather than a definitive conclusion. Future studies incorporating fibrosis-specific imaging or histopathological correlation are needed to confirm this mechanism.

Fourth, the current study did not measure soluble IL-6 receptor levels, TCZ serum concentrations, or pharmacodynamic markers of IL-6 pathway inhibition, limiting our ability to definitively confirm the molecular mechanism of IL-6 elevation at the receptor level. Fifth, the relatively short follow-up period (median 2.0 months) limits our ability to draw definitive conclusions regarding the long-term persistence of EOM regression. While our current findings illustrate the initial structural changes following TCZ therapy, evaluating the long-term durability of these anatomical outcomes remains an important direction for future research. Long-term outcomes are being collected in an ongoing extension study and will be reported separately.

## Conclusions

This study confirms that TCZ is a rapid, effective, and safe therapeutic intervention for active moderate-to-severe TAO. Using quantitative 3D mDixon-Quant MRI, this study provides objective evidence of the structural effects of TCZ on orbital tissues. Treatment selectively induces regression of hypertrophic EOMs, with the most pronounced reduction observed in the SR. In contrast, the MR shows relative resistance, likely attributable to early fibrosis-associated changes. Furthermore, the significant decline in serum TRAb levels underscores the systemic immunomodulatory effects of TCZ. Although the improvement in proptosis is clinically modest, the substantial reduction in orbital inflammation and short-term stability suggest that TCZ may serve as an effective strategy for rapid inflammatory control in TAO. Future large-scale prospective longitudinal studies are warranted to further evaluate the long-term persistence of these structural changes and their impact on recurrence rates.

## Data Availability

The original contributions presented in the study are included in the article/**Supplementary Material**. Further inquiries can be directed to the corresponding authors.
