# Supplementary material for: Selective extraocular muscle regression following tocilizumab in active thyroid associated ophthalmopathy: a 3D MRI-based retrospective study
Source: Front Endocrinol (Lausanne). 2026 Aug 26;17:1930592. doi: 10.3389/fendo.2026.1930592 (PMC13553465; doi:10.3389/fendo.2026.1930592)
Supplement: Supplementary file 1 [file SupplementaryFile1.docx]

**Supplementary Figure Legends**

**Supplementary Figure 1. Clinical and radiographic trajectory of Supplementary Case 1 (Steroid-Refractory).**

Serial nine-direction gaze photographs at T1 **(A)**, T2 **(B)**, and T3 **(C)**.

Longitudinal changes in CAS **(D)**, proptosis (mm) **(E)**, and IL-6 (pg/mL) **(F)**, illustrating inflammatory inactivation in a steroid-resistant patient over a 20-week period following TCZ treatment.

Coronal STIR MRI at T1 **(G)** and T2 **(H)**. Yellow arrows indicate marked reduction of inflammatory edema within the EOMs.

Three-dimensional orbital soft tissue reconstructions demonstrating regression of retrobulbar tissue at T1 **(I)** and T2 **(J)**, primarily driven by reduction of EOM hypertrophy.

**Supplementary Figure 2. Clinical and radiographic trajectory of Supplementary Case 2 (Primary Non-responder).**

Serial nine-direction gaze photographs at T1 **(A)**, T2 **(B)**, and T3 **(C)**.

Longitudinal changes in CAS **(D)**, proptosis (mm) (E), and IL-6 (pg/mL) **(F)**, illustrating limited clinical improvement and persistently elevated proptosis over a 20-week period following TCZ treatment.

Coronal STIR MRI at T1 **(G)** and T2 **(H)**. Imaging shows negligible changes in EOM CSA.

Three-dimensional orbital soft tissue reconstructions demonstrating minimal structural remodeling at T1 **(I)** and T2 **(J)**, consistent with clinical resistance.

**Supplementary Figure 3. Clinical and radiographic trajectory of Supplementary Case 3 (Clinical Relapse).**

Serial nine-direction gaze photographs at T1 **(A)**, T2 **(B)**, and T3 **(C)**.

Longitudinal changes in CAS **(D)**, proptosis (mm) **(E)**, and IL-6 (pg/mL) **(F)**, illustrating a transient therapeutic response followed by clinical relapse and symptomatic rebound after treatment cessation.

Coronal STIR MRI at T1 **(G)** and T2 **(H)**. Yellow arrows indicate reduction of inflammatory edema within the EOMs during the active treatment phase.

Three-dimensional orbital soft tissue reconstructions demonstrating structural regression from T1 **(I)** to T2 **(J)**, primarily driven by reduction of EOM hypertrophy during the treatment phase, despite subsequent clinical relapse.
