# Supplementary material for: Selective extraocular muscle regression following tocilizumab in active thyroid associated ophthalmopathy: a 3D MRI-based retrospective study
Source: Front Endocrinol (Lausanne). 2026 Aug 26;17:1930592. doi: 10.3389/fendo.2026.1930592 (PMC13553465; doi:10.3389/fendo.2026.1930592)
Supplement: Supplementary file 2 [file Table1.docx]

**Supplementary Table 1.** Longitudinal profiles of clinical, serological, and radiographic parameters in three representative therapeutic response scenarios.

| **Parameters** | **Supplementary Case 1**  **(****Steroid-Refractory)** | | | **Supplementary Case 2**  **(****Primary Non-responder)** | | | **Supplementary Case 3**  **(****Clinical Relapse)** | | |
| --- | --- | --- | --- | --- | --- | --- | --- | --- | --- |
| **Clinical Features and Phenotype** | Resistant to steroids, prompt relief of diplopia and proptosis with TCZ | | | Active-fibrotic overlap, persistent severe proptosis, minimal structural response | | | Transient remission followed by IL-6 surge and clinical rebound | | |
| **Time Point** | T1 | T2 | T3 | T1 | T2 | T3 | T1 | T2 | T3 |
| **CAS** | 3 | 0 | 0 | 3 | 2 | 3 | 3 | 0 | 3 |
| **Proptosis (mm)** | 21.00 | 19.00 | 19.00 | 35.00 | 33.00 | 33.50 | 16.00 | 16.00 | 17.00 |
| **IL-6 (pg/mL)** | 10.23 | 1.50 | 1.50 | 21.84 | 20.95 | 21.55 | 11.10 | 8.70 | 21.10 |
| **Orbital Tension** | 2 | 1 | 1 | 0 | 2 | 1 | 0 | 0 | 0 |
| **SR (mm2)** | 37.60 | 34.00 | N/A | 50.50 | 50.10 | N/A | 78.90 | 73.20 | N/A |
| **IR (mm2)** | 44.50 | 49.10 | N/A | 62.20 | 61.80 | N/A | 87.60 | 73.90 | N/A |
| **LR (mm2)** | 51.90 | 39.10 | N/A | 42.70 | 42.10 | N/A | 79.10 | 73.50 | N/A |
| **MR (mm2)** | 25.60 | 27.50 | N/A | 37.20 | 38.50 | N/A | 73.50 | 55.20 | N/A |
| **OFV (cm³)** | 17.80 | 17.50 | N/A | 33.60 | 29.80 | N/A | 21.80 | 22.90 | N/A |
| **TRAb (IU/L)** | 6.50 | 6.35 | 6.40 | 8.40 | 8.55 | 8.52 | 3.75 | 3.62 | 3.65 |
| **FT3 (pmol/L)** | 2.94 | 3.05 | 3.01 | 3.45 | 3.48 | 3.46 | 3.02 | 2.54 | 3.04 |
| **FT4 (pmol/L)** | 1.16 | 1.22 | 1.19 | 1.62 | 1.65 | 1.65 | 1.07 | 1.10 | 0.99 |
| **TSH (mIU/L)** | 2.29 | 3.88 | 3.85 | 2.15 | 2.18 | 2.16 | 0.04 | 0.83 | 1.33 |
